# Immune Age, Cardiovascular Disease, and Anti-Viral Immunity

**DOI:** 10.3390/cells14221793

**Published:** 2025-11-14

**Authors:** Kevin-Phu C. Le, Fahad Shuja, Jorg J. Goronzy, Cornelia M. Weyand

**Affiliations:** 1 Department of Medicine, Mayo Clinic, Rochester, MN 55905, USA; 2 Department of Immunology, Mayo Clinic, Rochester, MN 55905, USA; 3 Department of Cardiology, Mayo Clinic, Rochester, MN 55905, USA; 4 Division of Vascular and Endovascular Surgery, Department of Surgery, Mayo Clinic, Rochester, MN 55905, USA; shuja.fahad@mayo.edu; 5 Department of Medicine, School of Medicine, Stanford University, Stanford, CA 94305, USA

**Keywords:** T cell aging, B cell aging, macrophage aging, viral infection, cardiovascular disease, atherosclerosis, hematopoietic stem cell, DNA methylation, immune checkpoint

## Abstract

Cardiovascular morbidity and mortality rise precipitously during the 6th–9th decades of life, identifying aging as a critical risk factor. Simultaneously, older individuals are susceptible to severe viral infection, raising the question whether shared mechanisms exist that predispose to both cardiovascular disease (CVD) and failing anti-viral immunity. The aging process causes steady decline in immune fitness (immune aging), which undermines the ability to generate protective anti-viral immune responses. Paradoxically, the aging immune system supports unopposed inflammatory pathways (inflammaging), which exacerbates tissue inflammation in CVD, specifically atherosclerosis. Here, we review the current evidence of how innate and adaptive immune aging promotes tissue-destructive inflammation in atherosclerosis while failing to fight viral infections. Further, we consider how these two disease processes mutually influence each other. We propose that mounting an effective anti-viral response induces off-target bystander activation and exhausts immune cells, ultimately exacerbating CVD. Additionally, we explore how atherosclerotic CVD impacts innate immunity through epigenetic modification of hematopoietic precursors and metabolically conditioning immune cells, leading to a dysfunctional immune system that accelerates plaque inflammation while simultaneously impairing host defense.

## 1. Introduction

**Figure 1:**
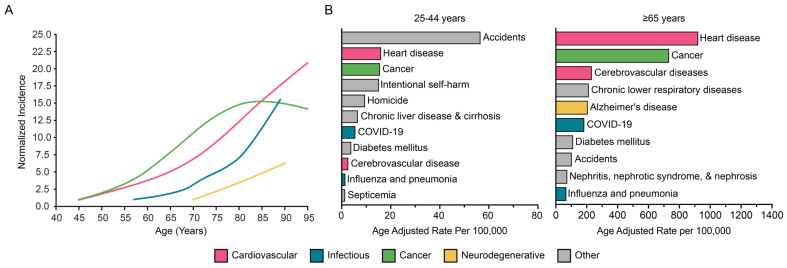
Age-associated morbidities. (**A**) Incidence of cardiovascular disease, influenza-related hospitalizations, cancer, and Alzheimer’s disease as a function of age. Modified plot from Rae et al. [3]. (**B**) Top ten leading causes of death in young (25–44 years) and older (≥65 years) individuals in the US (January 2018–February 2025).

Globally, the population of adults over the age of 65 is projected to nearly double by 2050, increasing from about 10% to 16% [1]. Twenty-three percent of the global total disability-adjusted life years (DALYs), a metric assessing a population’s overall disease burden, is attributed to adults aged 60 years and older. In high-income countries, older adults account for nearly 50% of this burden. CVD, neurodegeneration, and cancer account for a large proportion of this burden [2]. Indeed, the incidences of these chronic diseases and infectious disorders rise exponentially with age (Figure 1A) [3]. Whereas the leading causes of death in young adults aged 25–44 years include accidents and self-harm, death in older adults aged ≥65 years are mostly due to chronic diseases (e.g., CVD, cancer, neurodegeneration) and infection (e.g., influenza, COVID-19) (Figure 1B) [4].

In the United States, CVD prevalence doubles from the 40–59 age group to those 80 years or older, rising from around 40% to 80% of all men and women. CVD affects a multitude of vascular beds and manifests as myocardial infarction (MI), stroke, and peripheral arterial disease (PAD). Coronary artery disease (CAD) and stroke represent two-thirds of CVD deaths with one-third of MI occurring in those ≥75 years of age. Comparing adults 65–69 years old to those ≥85, PAD prevalence increases about 3- and 8-fold in men and women, respectively [1]. Approximately 80% of CVD-related deaths occur in those 65 or older, and CVD mortality nearly doubles when comparing 65–74 years olds to those older than 75 years [1]. Taken together, the drastic increase in CVD morbidity and mortality in older adults emphasizes that the aging process is the major risk factor for CVD. Mechanisms underlying age-related CVD risk range from aging-imposed structural changes in cardiovascular tissue to accumulative environmental exposure to inhalants and ingestants. A major risk determinant, however, lies in the reorganization of the immune system and how the aging process alters protective immune cells to damage-inducing effector cells.

The profound impact of aging on immune competence is exemplified by the inability of older adults to generate sufficient immune responses. A prototypic example is the infection with human cytomegalovirus (CMV), a common herpesvirus which escapes elimination and establishes chronic viral persistence [5]. In developed countries, >60% of adults are anti-CMV IgG positive, with the rate reaching 100% of the population in developing countries [5]. CMV is an opportunistic infection that rarely causes overt symptoms. However, under conditions of severe immunocompromise (e.g., AIDS, organ transplant), CMV may lead to organ damage [5]. CMV infection is associated with impaired survival in seropositive individuals aged 65 and older, resulting in a 3.7-year shortening in life expectancy [5]. CMV-infected individuals show a 2-fold increase in CVD-related death without increases in other causes [6]. While not clinically overt, older adults may reactivate CMV, indicating the need for sustained T cell immunity to keep the virus in check [7]. Declining control of latent infection in aged hosts extends to other herpesviruses, including varicella zoster (VZV) and Epstein–Barr virus [7,8]. VZV reactivation manifests as herpes zoster (HZ), also known as shingles. HZ risk increases significantly after the age of 50, leading to complications such as post-herpetic pain [8]. Poor viral infection outcomes in older adults are not exclusive to opportunistic and latent pathogens. Individuals ≥65 years of age show increased morbidity and mortality after infection with respiratory viruses, including influenza and SARS-CoV-2 [4,9,10]. Overall, older adults are susceptible to viral infection and viral reactivation, emphasizing that aging negatively impacts immune fitness and undermines successful anti-pathogen immunity.

**Figure 2:**
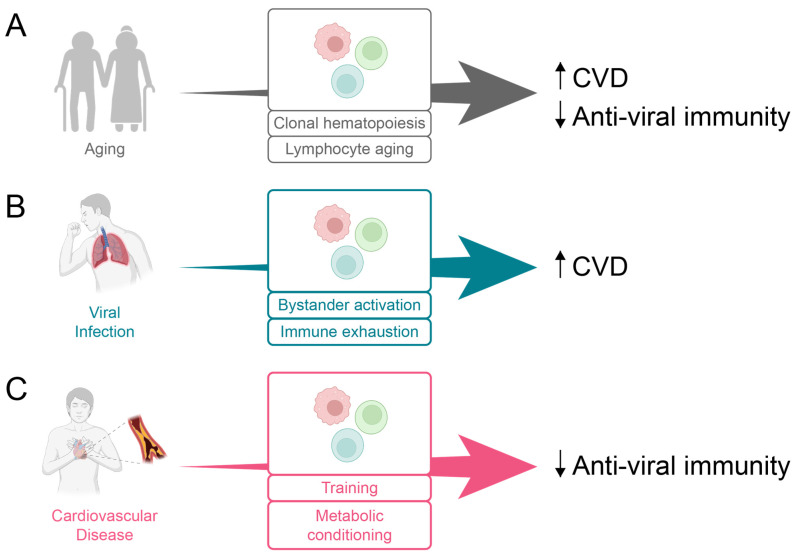
Mechanisms underlying the concomitant rise of infectious and cardiovascular disease. (**Model A**) Aging of myeloid cells and lymphocytes exacerbates cardiovascular disease while impairing viral defense. (**Model B**) Viral infection leads to an antigen non-specific bystander activation and immune exhaustion, together promoting cardiovascular disease. (**Model C**) Cardiometabolic disease reprograms innate and adaptive immunity, undermining anti-viral immune responses.

CVD and susceptibility to infection increase steadily with host age prompting us to explore whether there are shared mechanisms that both accelerate CVD and compromise anti-viral immunity (Figure 2). Although CVD encompasses multiple conditions, we focus on atherosclerotic disease and the following questions. Does insufficient protection against viral infection speed up atherosclerosis, the major process underlying CVD? Does atherosclerotic disease impair the ability to fight and eliminate viral pathogens? Here, we summarize the evidence linking CVD progression to weakened anti-microbial immunity, focusing on shared pathways in pathogenic immunity that contribute to both atherosclerosis and insufficient viral defense.

## 2. The Process of Immune Aging

Tissue inflammation, a primarily host-protective immune response, shields against pathogens and malignancy. Yet in the atherosclerotic plaque, tissue inflammation sustains and exacerbates disease [11]. Epidemiologic data support the concept that viral infections, particularly SARS-CoV-2 and influenza, accelerate risk for CVD [12,13]. To understand the connection between chronic plaque inflammation and host-protective anti-microbial immune responses, we first discuss how the immune system changes with age.

**T cells.** In humans, the thymus, the organ responsible for T cell development, begins to shrink as early as one year after birth. Thymic involution, evidenced by decreased parenchymal mass and fat accumulation, continues with age [14]. In the third decade of life, the thymus loses its dominant role as the T cell-producing organ. Accordingly, adults rely on post-thymic proliferation for new T cell generation and homeostatic maintenance [15]. Indeed, young adults derive around 84% of their total T cells from extra-thymic generation. In older adults, the thymus contributes to less than 1% of total T cells [15].

With progressive age, the naïve T cell compartment decreases in size as older adults fail to sustain naïve T cells against renewal pressure. Naïve CD4^+^ T cells decrease modestly, while naïve CD8^+^ T cells decline more drastically [16]. This shrinkage of naïve T cell populations occurs alongside an increase in end-differentiated T cells, so-called TEMRAs, effector memory T cells that re-express CD45RA. TEMRA expansion reflects chronic antigenic stimulation such as in the case of chronic CMV infection, and TEMRAs are typically clonally expanded. In addition to T cell subpopulation redistribution, the overall TCR diversity declines, leading to the characteristic TCR repertoire contraction in older adults [17,18]. Careful functional assessment of T cells in young and older adults shows a series of characteristic changes, all contributing to the state of immunodeficiency in older individuals (Table 1). Also, the TCR signaling machinery is less efficient in older T cells, reflecting age-dependent changes in intracellular signaling networks [19].

T cells are explicitly long-lived cells. In an animal model of repetitive antigenic restimulation, CD8^+^ T cells outlived the host, identifying them as potentially immortal [20]. Studies of monozygotic twins that shared the placenta during embryonic development indicate that T cells with identical TCRs persist even after 60–70 years [21]. However, the lifetime proliferative pressure imposed on T cells causes cumulative stress on the subcellular organelles that keep them alive and functional. In comparative studies of young and older T cells, T cell aging associated with the loss of mitochondrial and lysosomal competence [22,23]. In mice, selective loss of mitochondrial function in CD4^+^ T cells sufficiently induces an array of aging-related inflammatory diseases, including CVD [22]. Further, T cell aging is associated with lysosomal aging, which causes decreased handling of intracellular waste. As a result, the aged T cell relies on exosomes to expel waste, transforming it into a factory for waste production [23].

Much has been learned about T cell aging from studying age-associated inflammatory diseases such as rheumatoid arthritis (RA) [18,24,25]. In essence, RA patients prematurely age their immune system. Their T cells exhibit genome instability, mitochondrial deficits, lysosomal failure, and endoplasmic reticulum expansion (Table 1). Ultimately, the frailty of aging individuals is reflected at the cellular level, as evidenced by the compromised and dysfunctional subcellular organelles in their aging T cells.

Moreover, T cell aging translates into functional loss. Typically, older adults generate lower frequencies of memory T cells, disabling their ability to respond promptly to antigen re-exposure [15]. Also, persistent activation leads to a state of T cell exhaustion, leaving the host with T cells that have little or no proliferative capacity [26]. T cell exhaustion is best known as the mechanism underlying impaired anti-tumor immune responses in older individuals. Breaking T cell exhaustion is thought to be a critical mechanism through which checkpoint inhibitor therapy unleashes anti-tumor T cells, empowering the host to kill malignant cells, stop tumor growth and prevent metastasis [27].

An emerging field explores the degree to which T cells are affected by aging APCs. Antigen recognition is central to the induction of a successful adaptive immune response. T cells bind antigens presented on major histocompatibility complexes (MHCs), which ultimately determines their activation, differentiation, and expansion. Immune competence thus requires not only functional T cells but also effective APCs. Recent data suggest that age-induced deterioration of APCs profoundly influences the nature and duration of T cell responses. In individuals with the autoimmune vasculitis giant cell arteritis, dysfunctional APCs lead to autoreactive T cells responses [28,29,30]. Additionally, impaired APCs are responsible for insufficient anti-viral immune responses in individuals with CVD [31]. In both instances, aged APCs alter T cell responses, converting them from protective to disease-promoting.

**B cells.** In conjunction with T cells, B cells represent another arm of the adaptive immune system. They express B cell receptors (BCRs), and upon recognizing antigen, B cells activate, differentiate, and expand. Their key effector functions include antibody production, cytokine release, and antigen presentation [32]. During the aging process, B cells show characteristic adaptations (Table 1). There is agreement that total B cell number declines with age [33]. As with aged T cells, aged B cells lose efficiency in generating host protective responses. Typically, aged B cells exhibit contracted BCR repertoires, impaired memory response generation, and class switching defects [34].

In parallel to the age-related accumulation of TEMRAs, older adults accumulate age-associated B cells (ABCs). These ABCs do not readily respond to BCR ligation but instead show rapid response to toll-like receptor (TLR) ligation, specifically to the intracellularly located TLR7 and TLR9 [35]. Thus, ABCs lose antigen specificity and gain responsiveness to pattern recognition receptors (PRRs), a class of innate immune receptors that bind to pathogen- (PAMPs) and danger-associated molecular patterns (DAMPs). ABCs are thought to mostly derive from memory B cells that have seen antigen in the context of infection and vaccination. ABCs have recently been described to be regulated by a unique transcription factor profile and typically accumulate in patients with autoimmune disease [35]. They specialize in the production of autoantibodies and are considered critical effector cells in RA and systemic lupus erythematosus [35].

**Macrophages.** Bridging innate and adaptive immunity, macrophages (Mφ) serve as sentinels of the immune system. They are multifaceted effector cells, guiding immune responses that worsen tissue inflammation while also participating in tissue repair. Mφ exhibit multiple functions including cytokine and chemokine release, phagocytosis, antigen presentation, and lipid clearance. In inflammatory settings, Mφ release cytokines (e.g., IL-1β, IL-6, TNF) and present antigen to T cells, thereby sustaining the immune response. Conversely, as professional phagocytes, Mφ clear tissue debris, dead cells, and lipid deposits to re-establish tissue homeostasis [31].

Although these functions were initially ascribed to a dichotomous model describing pro-inflammatory M1 and anti-inflammatory M2 macrophages, recent studies have shed light on the incredible heterogeneity exhibited by macrophages across human tissues and disease states, revealing that many macrophage subsets express genes known to both sides of the M1/M2 spectrum. For example, a macrophage subpopulation characterized by high expression of osteopontin (*SPP1*) expresses genes associated with both M1 and M2 states, including pro-inflammatory mediators *CCL2* and *IL1B* and the scavenger receptor and lipid transporter *CD36* [36]. This *SPP1*^+^ macrophage is implicated in the pathogenesis of multiple diseases, including various malignancies [37], atrial fibrillation [38], and Alzheimer’s disease [39]. Additionally, recent efforts have even attempted to reclassify this *SPP1*^+^ macrophage, bringing attention to its shared phenotype of fibrosis and immunomodulation across pathologies, especially in aging-related diseases [36].

Moreover, while the impact of aging on lymphocyte function is well-understood, much less is known about how Mφ age. Because myeloid cells have short lifespans spanning hours to weeks, their aging process is primarily dominated by the aging of their progenitors, the hematopoietic stem cells (HSCs). During the aging process, somatic mutations accrue in HSCs. Older adults with hematopoietic mutations in *DNMT3A*, *TET2*, *ASXL1*, and *JAK2* show nearly double the CAD risk and a 4-fold increase in MI risk when compared to non-carriers [40]. Mutations in these genes are associated with clonal hematopoiesis of indeterminate potential (CHIP), a phenomenon describing HSC clonal enrichment in aged adults. These mutations are rare in adults under 40 years old but steadily increase in the elderly. Around 10% of adults 70 years or older and 18% of adults 90 years or older exhibit these somatic mutations, as defined by a ≥2% allele frequency in the peripheral blood [41]. In mice, *TET2* loss is sufficient to exacerbate atherosclerosis, confirming the causal link between CHIP and CVD [40,42].

Emerging evidence suggests that CHIP heightens the risk of hematologic cancer and infection-related complications in carriers. One recent meta-analysis described a 428% and 146% increase in risk of hematologic malignancy and severe COVID-19 in individuals with CHIP [43]. Moreover, CHIP-enriched mutations are also common in myeloid neoplasms such as acute myeloid leukemia and myelodysplastic syndromes. In CHIP carriers, the most common malignancies that develop are myeloid neoplasms [44]. Together, these observations suggest that mutations arising in HSC during the aging process induce immune dysfunction in older adults. One key element of CHIP is that certain myeloid clones gain a survival advantage. Rising evidence indicates that clonal populations directly contribute to disease, exacerbating cardiovascular pathology while simultaneously leading to immunosuppression.

Although still in its nascency, the field of Mφ aging has uncovered some data on subpopulation dynamics, providing opportunities to identify biomarkers. Circulating monocytes, the Mφ precursor cells, show no differences in number when comparing young versus older adults. However, older adults exhibit an enrichment of intermediate (CD14^+^ CD16^+^) and non-classical (CD14^+^ CD16^++^) monocytes [45]. Also, bone marrow cells positive for CD68, a Mφ lineage marker, decrease with age [46]. Like aging T cells and B cells, Mφ in older adults are functionally distinct from young myeloid cells (Table 1). As a common denominator, Mφ aging is associated with the process of inflammaging, a low-grade chronic inflammation in the aged adult. With increasing age, Mφ are characterized by a senescence-associated secretory phenotype (SASP), which includes high output of pro-inflammatory cytokines. Mφ aging causes changes in cell metabolism and organelle fitness, including impaired autophagy and mitochondrial dysfunction. Defective autophagy is linked to increase IL-1β release, and bioenergetic shifts may affect Mφ longevity and polarization states [47]. During aging, Mφ exhibit impaired phagocytosis, wound healing capacity, and migration [47,48]. Together, such deficiencies may inhibit resolution of chronic inflammation and impair the ability of the host to respond to foreign pathogens.

## 3. Viral Infection as a Risk Factor for Cardiovascular Disease

Epidemiologic data demonstrate that adults infected with respiratory viruses are at much higher risk for CVD. A landmark study yielded evidence that patients with acute lower respiratory infection face an increased risk of MI within the first three days of infection. Upon follow-up, MI risk in these individuals does not return to baseline for at least 3 months post-infection [49]. The association between infection and complications of CVD was confirmed in subsequent studies showing that adults positive for laboratory-confirmed influenza infection have a 6-fold increase in acute MI incidence when compared against non-infected controls [12]. This elevated CVD risk after viral infection extends to other respiratory viruses such as SARS-CoV-2. Even 30 days after infection, recovered individuals exhibit heightened risk of CVD spanning multiple disease categories including cerebrovascular disease, arrythmias, ischemic heart disease, and thromboembolic disease [13]. A later study corroborated the harmful effects of COVID-19 on cardiovascular outcomes and also reported a reduction in acute MI risk in vaccinated versus unvaccinated individuals [50]. Additional evidence connecting viral infection and CVD arises from studies exploring the impact of latent viral infections on CVD risk. Compared to non-infected individuals, those infected with human immunodeficiency virus (HIV) have a 1.5 to 2-fold increased risk of being diagnosed with clinically significant CVD [51]. Similarly, patients with chronic CMV infection are two-fold more likely to die from complications of CVD without increase in other causes [6].

While viral infection accelerates CVD risk, vaccination against pathogens appears to be host protective. Vaccination of older individuals against influenza reduces the risk of cardiac and cerebrovascular disease by 19% and 16–23%, respectively [52]. Similarly, a study conducted during the COVID-19 pandemic revealed that vaccination against SARS-CoV-2 reduced the frequency of acute MI and stroke [53]. This cardiovascular protection delivered by vaccines extends beyond non-viral microbes, as pneumococcal vaccination reduces CVD risk in adults 65 years and older [54].

**Figure 3:**
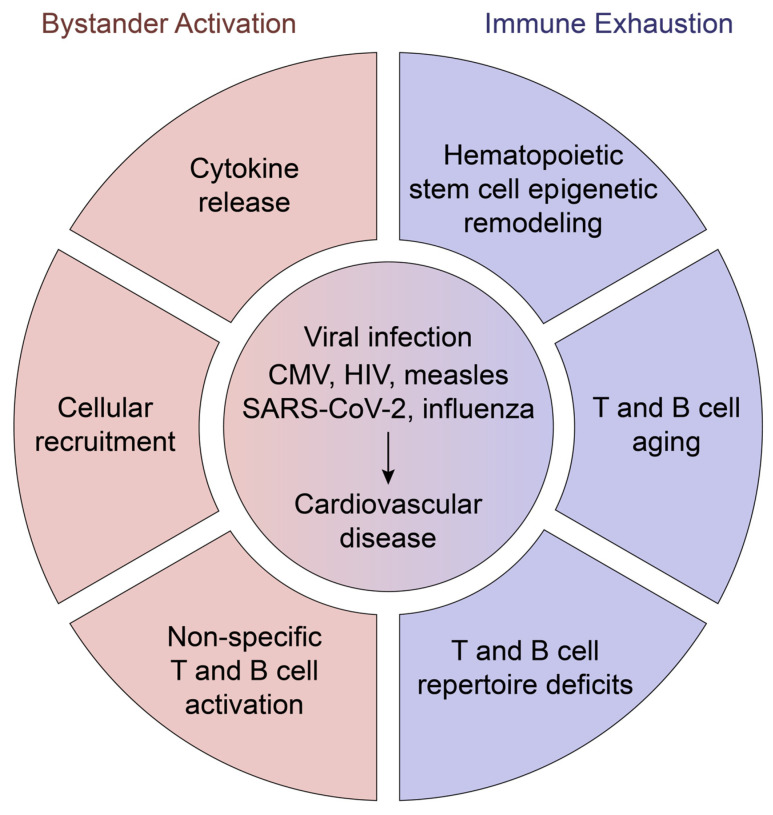
Anti-viral immunity exacerbates cardiovascular disease. Anti-viral immunity imprints the host immune system through an array of mechanisms, ultimately fostering the inflammation reaction that drives atherosclerosis.

The close association between viral infection and CVD incidence supports the notion that host defense against pathogenic microbes directly impacts cardiovascular disease processes. How can anti-viral immune responses influence the pathogenic immunity underlying CVD? Possible mechanisms include the broad immune activation in response to viral infection that elicits bystander inflammation. Further, chronic anti-viral immune responses affect the host by exhausting both innate and adaptive immune cells, redirecting immunity away from antigen-specific protection to poor and non-specific reactivity (Figure 3).

**Bystander Inflammation.** Response to viral invasion requires broad mobilization of innate and adaptive immune cells, ideally coupled with protective mechanisms that trigger contraction of immunity after antigen elimination. Early in the infection, Mφ recognize pathogen-derived products with their PRRs and initiate innate responses [55]. This initial reaction produces anti-viral (e.g., IFNɑ, IFNβ) and inflammatory cytokines (e.g., IL-1β, TNF), which can directly damage the vasculature and promote a pro-atherogenic environment [55,56]. Chemokine release by infected stromal cells recruits immune cells but may also enhance accumulation of responding cells in non-infected tissue. A key feature of innate immunity is a rapid, non-specific response involving the mobilization of first responder cells. Such first responders pave the way for the adaptive immune response carried out by antigen-specific T and B cells that can discriminate between self and non-self [55].

Ideally, anti-viral T cells would exclusively attack virus-infected cells, but activation and expansion of non-specific T cells also occur. Such bystander T cells respond to inflammatory cytokines and TLR ligands, eliminating antigen specificity as the sole selection agent. Typically, T cells infiltrating chronically inflamed tissue sites are polyclonal, illustrating that T cell responses are not exclusively directed against a triggering antigen. Bystander activation is typically considered as an underlying mechanism in autoimmune disease [57]. As recently noted, virus-specific CD8^+^ T cells accumulate in the atherosclerotic plaque, yet evidence is missing whether they encounter viral antigens in the tissue [58]. Studies comparing the repertoire of circulating and atheroma-residing T cells have demonstrated clonal sharing, specifically for end-differentiated T cells [59]. Notably, unstable atheromas harbor clonal TEMRA populations functionally biased towards high cytokine production [60,61]. Thus, immune cells infiltrating the atherosclerotic plaque are enriched for T cells with an aging phenotype, connecting T cell against plaque inflammation (Figure 3).

**Immune Imprinting, Exhaustion, and Senescence.** The atherosclerotic plaque is a non-healing wound, induced and sustained by the coordinated action of innate and adaptive immune cells that form chronic tissue infiltrates and mediate tissue damage. Recent clinical trials have validated the inflammation hypothesis, demonstrating that treatment with the anti-IL-1β antibody canakinumab or the anti-inflammatory drug colchicine significantly reduces cardiovascular events [62,63]. Notably, these broadly immunosuppressive interventions increase non-cardiovascular deaths, especially those secondary to infection. These unintended consequences indicate that anti-inflammatory cardiovascular event prevention sacrifices host defense. Increased pathogen susceptibility upon IL-1β suppression indicates that the same protective anti-microbial immunity also produces pathogenic inflammation. Mounting an effective anti-viral response, a process that creates and sustains inflammation, comes at the expense of accelerated inflammation in the vessel wall.

Viral clearance requires an efficient adaptive immune response, part of which entails T cell recognition of peptide:MHC complexes, activation, differentiation, and clonal expansion. Eventually, expanded clonotypes contract. Surviving memory T cells and B cells, with the capability to rapidly respond in case of re-exposure, sustain immune memory. It is now clear that infection imparts lasting changes on the immune system. CMV infection induces durable expansion of CD8^+^ T cells with persistent effector functions and effector memory phenotypes that last at least 4 years [64,65]. This phenomenon, termed memory inflation, is attributed to chronic low-level antigen stimulation from viral reactivation [64]. A recent study suggests that although acute infection selects for high-affinity TCR clones, chronic CMV infection enriches the TCR repertoire for those with low affinity [66]. Infection with human immunodeficiency virus (HIV), the virus species that leads to acquired immunodeficiency syndrome (AIDS), causes selective loss of CD4^+^ T cells, permanently reshaping the immune system [67]. Persistent viral infection with not only CMV and HIV but also EBV has been implicated in lasting expansion of T effector memory (T_EM_) and TEMRA cells [68,69].

The durable immunomodulatory effects stemming from antiviral defense extend to acute infections as well. Measles infection leads to a prolonged 2- to 3-year immunosuppression, predisposing to non-measles infectious disease mortality [70]. This immunosuppression clearly outlasts the infection itself, which only lasts days to weeks. Indeed, a prospective cohort study over a 20-year follow-up period showed that compared to those without measles infection, men with a prior history of measles infection exhibited an 8% lower total CVD mortality. Such results indicate how an acute infection imposes lasting effects that ultimately impact CVD risk [71]. Subsequent studies revealed that measles virus infection results in the elimination of 11–73% of the antibody repertoire in infected individuals, thereby increasing their susceptibility to future infection [72]. Related is a phenomenon known as original antigenic sin, implying that previous exposure to an antigen may impair the immune system’s ability to respond effectively to similar antigens in the future. In this model, B and T cells clonally expand to an initial serotype of virus such as influenza or dengue. Upon viral infection with a related serotype, the memory cells formed against the initial serotype, albeit ineffective against the second, expand and lead to impaired host defense [73]. Indeed, pre-existing immunity to influenza, including childhood infection, shapes future immune responses throughout life [74,75]. Even the route of exposure appears to influence subsequent immune responses: influenza vaccine-induced antibodies bind neutralizing epitopes, while infection-induced antibodies typically react to non-neutralizing ones [74].

Prior infections impact not only the adaptive immune compartment but also exert a profound influence on innate immunity. Recent studies indicate that infection induces epigenetic remodeling in innate cell populations, particularly myeloid cells. In a landmark study, healthy individuals who recovered from mild non-hospitalized COVID-19 exhibited antigen-agnostic shifts in both innate and adaptive immunity when compared against non-infected controls. In males, prior SARS-CoV-2 infection elevated baseline T cell activation and depressed monocytes innate immune genes including TLRs, which influenced responses to a challenge by a different antigenic stimulus–influenza vaccination [76]. Epigenetic rewiring in response to severe COVID-19 induces hyperactivated monocytes that upon stimulation, upregulate pro-inflammatory and antigen presentation programs [77]. Similarly, vaccination against influenza induces durable monocyte epigenetic signatures that temper responsiveness to TLR stimulation while increasing interferon responses against unrelated dengue and zika viruses. These outcomes persist for at least 180 days after vaccination, suggesting that vaccination not only protects against infection but may also mitigate future harmful inflammatory reactions [78]. Given the short-lived nature of monocytes, such epigenetic changes are thought to result, at least partially, from the reprogramming of HSCs in response to pro-inflammatory cytokines released during infection [77]. Interestingly, a form of innate immune memory also develops in tissue-resident Mφ, as shown by the enhanced interferon responses to influenza challenge in alveolar Mφ after respiratory viral infection [79,80].

Mechanisms underlying how viral infection-induced immune dysfunction affects CVD risk are expected to be pathogen specific. Worsened CVD may occur due to the effect to the memory inflation caused by CMV, leading to TEMRA expansion [68]. Because of their decreased proliferative capacity and increased inflammatory cytokine production, these T cells are considered to be in a senescence-like state and are thought to contribute to age-related chronic diseases [15]. It has also been proposed that CMV facilitates the increase in cardiovascular complications in individuals with HIV [81]. Considering that chronic HIV infection is recognized as a driver of immunosenescence, this mechanism would implicate immune system exhaustion in enabling tissue-damaging tissue inflammation in the cardiovascular system. In the evolving field of innate immune memory, however, a notable gap exists in current knowledge. There is a lack of studies exploring how virus-induced epigenetic remodeling impacts vascular disease and which mechanisms drive this process. Taken together, viral infections durably imprint the immune system, influencing the nature, trajectory, and durability of future immune responses (Figure 3). For many viral infections, immune system activation and exhaustion ultimately lead to increased cardiovascular burden.

## 4. Cardiovascular Disease as a Risk Factor for Viral Infection

**Figure 4:**
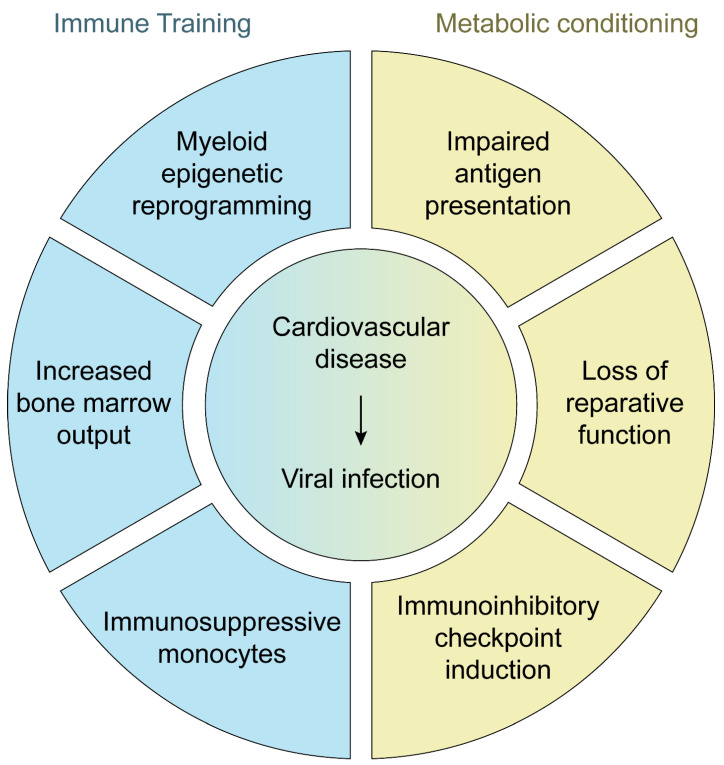
Cardiovascular disease compromises host defense. Cardiometabolic disease rewires the host immune system, diminishing anti-pathogen immunity through multiple pathways.

The relationship between the immune and the cardiovascular systems is not a one-way street. Instead, pre-existent CVD modifies immune competence, impairing overall immune fitness (Figure 4). Strong support for CVD-imposed immunomodulation derives from the COVID-19 pandemic, where individuals with pre-existing CVD suffered increased complications and consequentially poor outcomes from SARS-CoV-2 infection. Early data indicated that case fatality rates (CFR) of COVID-19 in patients with CVD (10.5%) far exceeded the overall CFR (2.3%) [82]. Later data confirmed the harmful effects of CVD comorbidity. Odds of fatal outcomes from COVID-19 increased by 155% in individuals with CVD when compared to those without. Age stratification revealed that risk of fatal outcomes increased most in younger populations. Individuals <50 years had an odds ratio (OR) of 5.66, much higher than the OR of 2.10 in those 60 years and older [83].

Defective immunity stemming from underlying CVD not only increases risk of severe outcomes from SARS-CoV-2 but also to other viral infections. Pre-existing CVD heightens the risk for complications and adverse outcomes after influenza infection. Individuals with CAD hospitalized for influenza exhibit 75% and 18% increased risk of infection-associated ischemic heart disease and heart failure, respectively [84]. The US CDC recognizes the heightened vulnerability of individuals with CVD to influenza-related complications and recommends prioritizing vaccination for this population [85]. Individuals with CAD also mount poor immune responses to chronic viral infection as evidenced by an 11–22% increased odds of HZ when compared to those without CAD [86].

Influenza vaccination, on the other hand, imparts protection against viral infection, subsequent complications, and infection-related death. In adults aged 65 and older, high-dose influenza vaccination proved 24.2% more effective in preventing laboratory-confirmed influenza infection when compared against the low-dose formulation [87]. However, high-dose vaccine does not provide additional host protection against cardiovascular complications. In individuals with high-risk CVD, high-dose influenza vaccination does not decrease all-cause mortality or cardiopulmonary hospitalizations when compared to the standard-dose vaccination [88]. Overall, these results suggest that CVD itself impairs the immune system, preventing the mobilization of an effective anti-viral immune response and raising the question of which molecular mechanisms drive the immunosuppressive effects of CVD. One hypothesis lies in immunomodulatory effects imposed by the vascular system and the inflammatory lesions underlying CVD. Age-related structural changes in the cardiovascular system and inflammatory mediators released by CVD lesions may re-educate immune cells through the process of trained immunity [89]. In this model, inflammatory and metabolic signals impose epigenetic modifications of HSC and their offspring, imparting lasting effects on innate immune cells. A second explanation stems from the metabolic co-morbidities prevalent in CVD. By shaping myeloid cell and lymphocyte differentiation and function, cardiometabolic factors leave an enduring imprint on the immune system and fundamentally alter host immunity.

**Immune Training**. The atherosclerotic plaque represents a tissue microenvironment that imposes systemic effects through pro-inflammatory mediator release [90]. Immune cells, predominantly T cells and Mφ, constitute the largest cell population in the plaque [91]. Sustaining this local plaque inflammation requires production of chemokines and inflammatory cytokines, which facilitates cell recruitment and activation. These molecules provide system-wide communication from the tissue microenvironment, resulting in a feedback loop that ultimately exacerbates the lesion. Other mediators (e.g., exosomes, microvesicles, metabolites, tissue fragments) may serve similar functions and imprint the host immune system.

IL-1β, a prototypical inflammatory cytokine implicated in exacerbating atherosclerotic lesions [62], can reprogram bone marrow HSC. Indeed, β-glucan-induced HSC epigenetic remodeling and the subsequent myelopoietic expansion is mediated in part by IL-1β [92]. Interestingly, anti-IL-1β treatment itself restricts HSC differentiation to the myeloid lineage while also impairing HSC self-renewal capacity [93]. This cytokine-mediated HSC conditioning extends to other inflammatory molecules including IL-6. A recent study confirmed that after SARS-CoV-2 infection, IL-6-exposed human HSC displayed durable epigenetic changes, which affected downstream monocyte TLR responses [77]. Given their role as progenitor cells, epigenetic modifications in HSC will result in substantial impact on descendent cells such as monocytes and Mφ. Taken together, systemic inflammatory mediators affect hematopoietic progenitors, thereby facilitating lasting changes in daughter cells, specifically those of the myeloid lineage.

HSC imprinting and the subsequent change in downstream descendants relates to the concept of trained immunity, which describes how epigenetic modifications triggered by a primary stimulus can lead to durable future effects. This effect, sometimes called innate memory, however, differs from adaptive memory in duration and specificity. Adaptive memory is antigen-specific and, as long as memory T cells and B cell remain alive, adaptive memory can last a lifetime [94]. Although myeloid cells live only hours to days, HSC imprinting provides a mechanism to impact future myeloid offspring responses. Indeed, trained myeloid cells react with heightened immune responses upon secondary challenge [89]. Although initially described as a response to β-glucan, trained immunity is now recognized to stem from a variety of stimuli, including oxidized low-density lipoprotein (oxLDL), hypocretin, and IL-1β itself. IL-1β may act as a common downstream effector molecule that mediates the training [89]. A landmark study demonstrated that pre-exposure of monocytes to oxLDL, a prototypical lipid stimulus important in the context of atherosclerosis, led to heightened responses upon restimulation as evidenced by increased pro-inflammatory cytokine production and foam cell differentiation [95]. Interestingly, a follow-up study indicated that statin treatment did not reverse the effect of trained immunity on monocytes derived from patients with familial hypercholesterolemia, indicating that long-term exposure to lipids may impose durable epigenetic changes to innate immunity despite clinical intervention [96]. Moreover, a healthy volunteer trial employing the Bacillus Calmette-Guérin (BCG) vaccination validated the concept of immune training in humans. When treated with unrelated pathogens, peripheral blood mononuclear cells isolated from BCG-treated individuals produced a 2-fold higher amount of inflammatory cytokines such as IL-1β and TNF [97].

Although trained immunity can exacerbate inflammation, rising evidence indicates that innate immune remodeling in the bone marrow may also dampen immune responses. In a mouse model, IL-4Rɑ knockout in myeloid progenitors but not mature cells led to reduced tumor burden. Also, patients later treated with anti-IL-4Rɑ had decreased circulating monocytes but enhanced response to PD-1 checkpoint blockade [98], compatible with the concept that bone marrow reprogramming can lead to systemic immunosuppression.

Finally, cardiovascular events themselves can induce immune reprogramming, ultimately leading to immunosuppression and poor cancer outcomes [99]. One seminal study illustrated that MI accelerates breast cancer progression by reprogramming monocytes to an immunosuppressive phenotype while increasing their recruitment to the tissue. Further, breast cancer patients with pre-existing CVD exhibit 59% and 60% increased risk of recurrence and cancer-related mortality, respectively [100]. Overall, these results provide evidence that CVD modifies the innate immune epigenome, impairing host defense to future immune challenges.

**Metabolic Conditioning.** Dating back to 1961, one of the landmark Framingham Heart studies identified serum cholesterol as a significant risk factor for CAD, establishing the connection between dyslipidemia and CVD [101]. Recognizing that many lipid species contribute to cardiovascular risk, current therapies target not only serum LDL cholesterol but also non-HDL cholesterol and triglyceride levels [102]. Lipids, moreover, appear to impact disease processes outside of increasing CVD risk. Indeed, epidemiologic studies have demonstrated an association between dyslipidemia and the susceptibility to infection. A significant association exists between pneumonia hospitalization risk, low serum high-density lipoprotein cholesterol (HDL-C), and high triglyceride levels [103].

**Figure 5:**
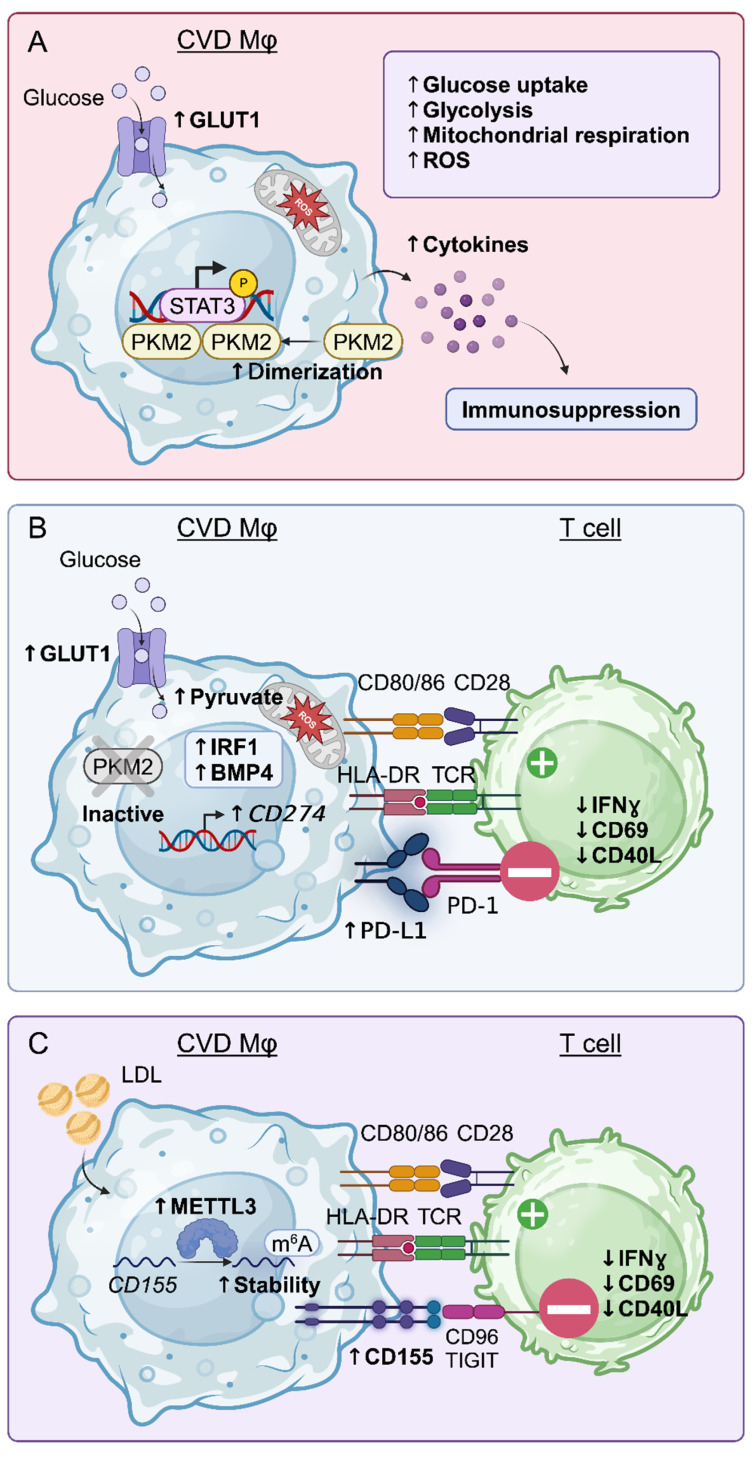
Cardiovascular disease metabolically conditions macrophages, leading to impaired immune responses. (**A**) Mφ from individuals with CVD have high glucose uptake, glycolysis, and mitochondrial activity. Increased reactive oxygen species (ROS) induce dimerization and nuclear translocation of the enzyme PKM2, which phosphorylates STAT3. These rewired Mφ increase cytokine production, thereby facilitating immunosuppression. (**B**) Glucose utilization by CVD Mφ controls PD-L1 expression. Elevation of intracellular pyruvate enhances *CD274* (*PD-L1*) expression. PD-L1^+^ Mφ provide negative signals to T cells via crosslinking with the PD-1 receptor. (**C**) CVD Mφ sense low-density lipoprotein (LDL), which induces expression of METTL3 a methyltransferase. METTL3 stabilizes *PVR* (*CD155*) mRNA through adenosine methylation, ultimately increasing surface expression of the immunoinhibitory ligand CD155. Upon binding CD96 and TIGIT, CD155 sends stop signals and paralyzes T cell immunity. The green pluses and red minuses represent positive activation and negative inhibitory signaling, respectively. Arrows indicate change in phenotype or protein expression.

Emerging data provide a molecular basis of how metabolic conditions redirect cellular function to impair immune fitness. Treating human Mφ with oxLDL, a major lipid component of the atherosclerotic plaque, causes downregulation of MHC II molecules such as *HLA-DRA*, *HLA-DRB1*, *HLA-DQA1* [104]. Thus, lipid conditioning adversely affects the ability of Mφ to effectively present antigen, thereby impairing the adaptive immune response. Recent single-cell RNA sequencing (scRNA-seq) studies confirm that lipid-associated Mφ exhibit markedly low expression of the antigen machinery when compared to other plaque Mφ subpopulations [105]. Supporting evidence for a direct immunomodulatory effect of CVD on immunocompetence comes from a landmark study illustrating that Mφ and monocytes derived from individuals with CAD exhibit profound metabolic rewiring, as evidenced by increased glycolytic flux and mitochondrial respiration (Figure 5A). Mechanistically, such bioenergetic changes promoted pyruvate kinase M2 dimerization and nuclear translocation, which induced a STAT3-mediated transcriptional program [106]. In support, Mφ and monocytes derived from individuals with CAD suppress CD4^+^ T cell activation (Figure 5C) [31]. This study, which focused on immune checkpoint molecules in individuals with CAD, found that CAD Mφ express high levels of the immunoinhibitory molecule CD155, which binds CD96 and TIGIT expressed on CD4^+^ T cells and sends stop signals to the interacting T cell. Mφ derived from individuals with CAD expressed high levels of the enzyme METTL3, a methyltransferase that modifies mRNA by adding a methyl group to adenosine residues. This modification has profound impact on mRNA stability, decay, and translation [107]. The heightened METTL3 levels in CAD Mφ stabilized *PVR* (*CD155*) mRNA. As a result, these Mφ produced increased CD155 protein, transforming them into immunoinhibitory cells. In search for the signals controlling the expression and function of METTL3, the authors screened for abundant metabolites in CAD patients. Treatment with LDL, oxLDL, or LDL-rich human plasma but not β-glucan, a known agonist of trained immunity, induced *METTL3* and *PVR* mRNA in monocytes [31].

In addition to dyslipidemia, dysglycemia is considered a long-term risk factor for CVD. Classically, hyperglycemia is associated with the metabolic and endocrine disorder diabetes mellitus (DM). Individuals with type 2 DM show a 2- to 4-fold increase in developing CVD [108]. Interestingly, regardless of DM status, high plasma glucose levels in patients admitted with MI are associated with significantly decreased long-term survival. An 18 mg/dL increase in glucose level raises the mortality risk by 4% and 5% in non-diabetic and diabetic individuals, respectively [109].

There is now evidence that glucose imprints immunity, functioning as a regulator of myeloid cells and consequently, the innate immune system. Mφ derived from individuals with CAD express higher levels of the immunoinhibitory molecule PD-L1 when compared to those of healthy donors (Figure 5B) [110]. Mechanistically, CAD Mφ exhibit inactive PKM2, which leads to increased mitochondrial pyruvate and heightened production of reactive oxygen species (ROS). Ultimately, this induces expression of BMP4 and IRF1, ultimately leading to increased PD-L1 expression [110]. PD-L1 is a well-established regulator of anti-tumor immunity [111]. By upregulating PD-L1 expression, tumor cells escape tumor-specific T cells killing. Indeed, therapeutic strategies that disrupt PD-L1/PD-1 interactions revolutionized cancer immunotherapy.

Notably, expression of PD-L1 on CAD Mφ is directly controlled by glucose, placing metabolic signals upstream of immune function. CAD Mφ highly express glucose transporter 1 (GLUT1), allowing efficient import and subsequent metabolism of extracellular glucose [112]. Imposing glucose deficiency on CAD Mφ leads to downregulation of *SLC2A1* (*GLUT1*) mRNA expression, suggesting that glucose conditioning controls Mφ phenotypes through transcriptional regulation. Additionally, long-term high glucose conditions shape several functional domains of Mφ. Mφ exposed to a 7-day trial of high glucose exhibit reduced phagocytosis, decreased bactericidal ability, and lower nitric oxide generation when compared to their normal glucose counterparts. Also, these long-term glucose-conditioned Mφ produce higher amounts of the anti-inflammatory cytokine IL-10 [113]. Collectively, these results indicate that cardiovascular pathologies and their associated metabolic imbalances condition innate immunity. Such imprinting results in immunosuppressive cellular phenotypes, predisposing the host to infection, cancer, and tissue degenerative disease.

## 5. Conclusions

During the second half of life, the risk of mortality due to infectious, neurodegenerative, neoplastic, and cardiovascular diseases rises dramatically. The common thread underlying these diseases is the loss of a functional immune system. The aging immune system loses the ability to generate protective immune responses but gains inflammatory activity, often described as inflammaging. With diminishing protective immunity, the immune system’s ability to repair tissue also declines. Inflammatory conditions, such as Alzheimer’s disease and CVD reflect the inability of older adults to maintain tissue integrity. Susceptibility to cancer and infection mainly stems from the progressive failure of the adaptive immune system to generate highly specific T cell and B cell responses and to secure lasting immune memory. Here, we propose mechanisms that explain the concomitant and perplexing rise of CVD and infection susceptibility in older adults.

**Immune Aging.** With advancing age, T and B cells become less functional, diverse, and specific. In parallel, myeloid cells that function as first responders undergo profound changes, driven by clonal hematopoiesis and the lifelong exposure to internal and external stimuli, termed the gero-exposome. Collectively, this results in a state of immunodeficiency in which nonspecific, relatively primitive inflammatory responses outweigh the efficiency of adaptive immunity. Chronic tissue inflammation drives CVD, specifically the persistent smoldering vessel wall inflammation encountered in atherosclerotic lesions. Host infection with any viral pathogen elicits short-lived, intense immunity, which enhances tissue damage while undermining effective pathogen removal.

**Vascular Tissue Aging**. Over a lifetime, the vascular system is exposed to high mechanical stress. Collagen and elastic fibers, the major components of the vessel wall, have low turnover and are susceptible to fragmentation and accumulation of advanced glycation end products. Aging is associated with vascular stiffening and elasticity loss, which may arise from inappropriate immune responses towards vascular tissue. Indeed, a recent landmark study characterizing the human proteome across fifty years indicated that blood vessels exhibit early susceptibility to aging [114]. In turn, resulting inflammatory lesions profoundly impact the immune system itself. Chronic immune stimulation leads to immune exhaustion, which affects both the innate and adaptive arms of immunity. Circulating inflammatory mediators imprint bone marrow stem cells through epigenetic modifications, altering the generation and fate of myeloid cells. Ultimately, individuals with CVD are less competent at fighting pathogens, which requires highly efficient and swift immune responses.

**Immunometabolism**. The emerging field of immunometabolism captures the intimate relationship between metabolism and immune function. Immune cells are highly dependent on bioenergetic supplies, and conversely, metabolites are powerful regulators of immunity. Inflammation is an energy demanding process, requiring the consumption of bioenergetic and biosynthetic resources to produce new cells and cell resources (e.g., cytokines, enzymes, and vesicles). To generate tissue inflammation, T cells and Mφ require high nutritional supply. Inevitably, metabolic checkpoints control T cell function and fate [115]. In the autoimmune disease rheumatoid arthritis, T cells show signs of metabolic failure, which redirects effector functions and turns protective T cells into pathogenic T cells [116]. Indeed, metabolic rewiring is connected closely with T cell aging [117]. The aging T cell is pro-inflammatory, stemming from mitochondrial failure [118,119,120,121,122].

Ultimately, metabolic dysregulation is an integral part of CVD risk. Abnormalities in glucose and lipid metabolism are strong risk factors, rendering individuals susceptible to morbid manifestations of CVD. These metabolic abnormalities potently modulate immune cell function, as adaptive and innate immune cells are dependent on metabolic resources. These cells employ integrated programs that allow them to adapt to metabolite fluctuations. Metabolic signals, through a variety of molecular mechanisms, reprogram myeloid cells and their precursors, specifically, hematopoietic stem cells in the bone marrow. Metabolic signals determine T and B cell responses by modulating survival and differentiation. In essence, the aging immune system, shaped by the metabolic deviations of cardiometabolic disease, undergoes adaptations characterized by high inflammatory propensity and low antigen specificity.

## Figures and Tables

**Table 1:** Hallmarks of Immune Aging.

T Cell	B Cell	Macrophage
Thymic involution	Decreased BCR diversity	Clonal hematopoiesis
Naïve population decline	Impaired memory responses	Increased SASP
TEMRA expansion	Class switch recombination defects	Inflammatory cytokine production
Repertoire contraction	ABC enrichment	Reduced autophagy
Impaired TCR signaling	Heightened TLR ligand reactivity	Mitochondrial dysfunction
Genomic instability	Autoantibody production	Impaired phagocytosis
Poor mitochondrial fitness		Reduced reparative functions
Lysosomal dysfunction		
Proteostatic failure		
Impaired autophagy		
Exhaustion		

## Data Availability

No new data were created or analyzed in this study. Data sharing is not applicable to this article.
